# Immediate Implants in Extraction Sockets with Deficient Buccal Walls in the Maxillary Aesthetic Zone

**DOI:** 10.3390/dj13050185

**Published:** 2025-04-24

**Authors:** Sorin Boeriu, Timothy Hottel, Cris Chirla, Phillip Chirla

**Affiliations:** 1 Private Practice, Kitchener, ON, Canada; sorin.boeriu@gmail.com; 2 Comprehensive Care, School of Dental Medicine, Case Western Reserve University, Cleveland, OH 44106, USA; 3 Private Practice, Akron, OH 44312, USA; ccherila@gmail.com (C.C.); pchirla@gmail.com (P.C.)

**Keywords:** immediate implant, esthetic zone, fenestration, dehiscence

## Abstract

**Background:** Immediate implant placement in fresh extraction sockets has become an accepted treatment in dentistry as a predictable procedure to restore failing teeth. One prerequisite for this immediate procedure in the anterior maxillary region is an intact facial wall. Unfortunately, the presence of fenestrations and dehiscences is very common. These defects occur due to the pathology responsible for the extraction of the teeth. Traditionally, hard and soft tissue grafting is necessary to repair these large bony defects before implant placement. However, there are many defects with facial wall deficiencies. **Methods:** This report reflects procedures used to provide successful functional outcomes using grafting techniques in conjunction with immediate implant placement in defective sockets. This clinical research study followed a qualitative methodology, and the results are based on observational outcomes of four patient surgical implant procedures. Each patient received the same protocol in an attempt to reach similar results. **Results:** Proper diagnosis, treatment planning, and clinical skills are key factors in achieving predictable results. With each of these four patients, the clinical soft tissue outcomes revealed that the midfacial gingival margin had minimal or no recession at two years with minimal pocket depths less than 3 mm. **Conclusions:** Although the procedure presented in this article has yet to be clinically validated, it is an available technique that can be used in the hands of an experienced practitioner and can provide excellent results for the patient.

## 1. Manuscript

Tooth extraction is a very common occurrence in the dental office [1]. Depending upon the clinical conditions of the extraction socket and the alveolar ridge remodeling that occurs after extraction, different treatment protocols have been proposed [2,3,4,5].

The presence of an intact facial wall is considered a prerequisite for immediate implant placement. According to current scientific opinion, loss of the facial wall constitutes a contraindication for immediate implant placement (IIP), and a socket preservation technique or delayed implant placement should be used [6]. The 2019 consensus report on management of the extraction socket and timing of implant placement recommends that immediate implants should be avoided in extraction sites with severely damaged sockets (more than 50% loss of one or more walls) because the procedure may be associated with a higher risk of achieving unfavorable therapeutic outcomes [6].

According to Chen and Darby, the presence of facial wall defects (fenestrations and dehiscences) after a tooth extraction is very common. Immediately after an extraction, an intact buccal wall is present in only 47% of the cases. Facial bone defects are a common occurrence at extracted maxillary central and lateral incisor sites, with 53% of sites presenting with either fenestration or dehiscence defects [7]. Their study confirmed that teeth with failed endodontic therapy tended to present with fenestration defects, whereas teeth with vertical root fractures usually presented with dehiscence defects of the facial socket wall. A high proportion of teeth that were extracted due to the loss of crown retention and/or structural integrity of the remaining tooth, as well as teeth with external root resorption, had mainly intact socket walls [7]. Determining the etiology of the extraction plays a critical role in the prognosis of the future treatment: extraction with an etiology of excessive caries, root fractures, and endodontic failures has a better prognosis compared to tooth loss due to perio-endodontic lesions or severe periodontitis [1].

Implant placement in sites with periapical pathology has been extensively documented in the literature. Current evidence indicates that placement of immediate dental implants in extraction sites with chronic periapical infections can be successful, provided appropriate clinical procedures are performed to debride the socket prior to implant placement [8,9,10,11]. The most recent ITI Consensus report confirmed that the immediate implant protocol can be selected for teeth presenting with chronic periapical infections only when the following conditions exist: absence of fistula, infection can be completely debrided, and there is sufficient apical and palatal bone to provide primary implant stability and to place the implant in the correct three-dimensional (3D) position for restoration [12]. The disadvantage of the placement of implants into the sockets of teeth with periapical lesions is the potential for implant contamination during the initial healing period because of remnants of the infection [9]. However, the high success rate of fresh-socket implants placed in chronic and acute lesions may be explained by the behavior of endodontic infections because they are mixed infections dominated by anaerobic bacteria (*Fusobacterium*, *Prevotella*, *Porphyromonas*, *Actinomyces*, *Streptococcus*, and *Peptostreptococcus*) commonly restricted to the infected root canal [13]. Extraction of the involved tooth generally leads to eradication of the cultured microorganisms [14]. Furthermore, the presence of periapical pathology and a facial wall deficiency pose other clinical problems that must be considered if an immediate implant is to be placed at the time of the extraction or a delayed approach is preferred. One of the problems in IIP in chronically infected sites is the lack of bone in the apical and palatal area. It was recommended that a minimum residual apical bone of 3 to 5 mm in a vertical dimension is required [15]. The other factor that we have to consider is that alveolar ridge remodeling that occurs immediately after the extraction is influenced by the presence or absence of a facial wall defect, and therefore a reconstruction of the facial wall is required when immediate implants are placed in sites with facial wall deficiency [7].

Guided bone regeneration (GBR) techniques have helped eliminate the concerns surrounding bone deficiencies and allow implant placement in the correct 3D restorative-driven protocol. If a large bony defect is present after extraction, hard and soft tissue grafting is recommended, followed by a delayed implant placement protocol [9,10]. However, there are many studies reporting a favorable aesthetic outcome when placing implants in fresh extraction sockets with a buccal wall deficiency [9,11,12]. The purpose of this four-patient series is to provide a clinical, surgical protocol for the placement of immediate implants with a GBR protocol in sites with a large facial wall deficiency. 

## 2. Patient 1

**Figure 1:**
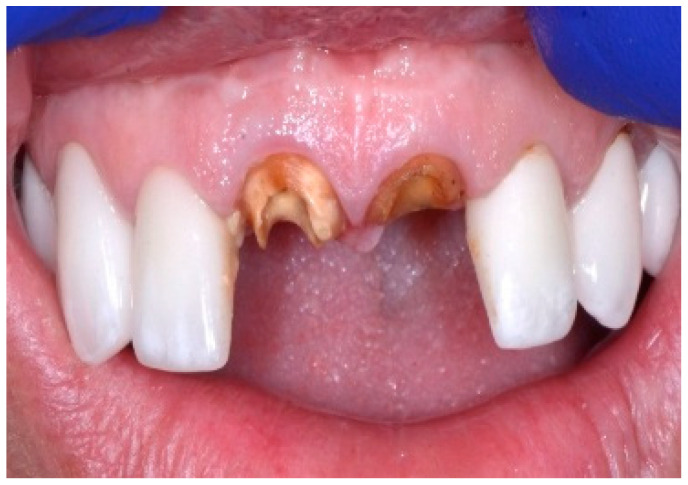
Clinical view of existing maxillary incisors.

A 43-year-old female patient in good health and a non-smoker presented with a history of endodontic failure of the maxillary central incisors. She was interested in the replacement of her teeth with endosseous implants. The teeth had previously been treated endodontically with no lesion regression. Endodontic surgery had also failed. Clinical examination revealed class 1 mobility and a scar on the soft tissue above the maxillary anterior teeth. Radiographic examinations revealed the presence of chronic periapical lesions associated with teeth number 8 and 9, and clinically there was minimal remaining tooth structure (Figure 1). A treatment plan involving the placement of two immediate implants followed by future ceramic crowns was presented and accepted by the patient.

### 2.1. Surgical Protocol 

**Figure 2:**
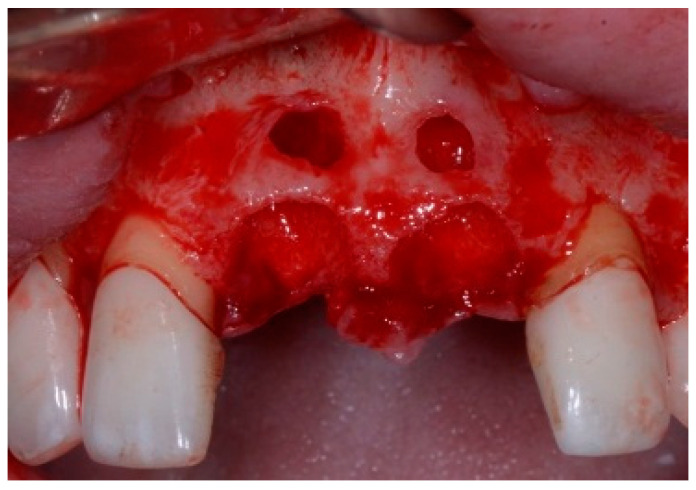
Clinical view immediately after extraction.

**Figure 3:**
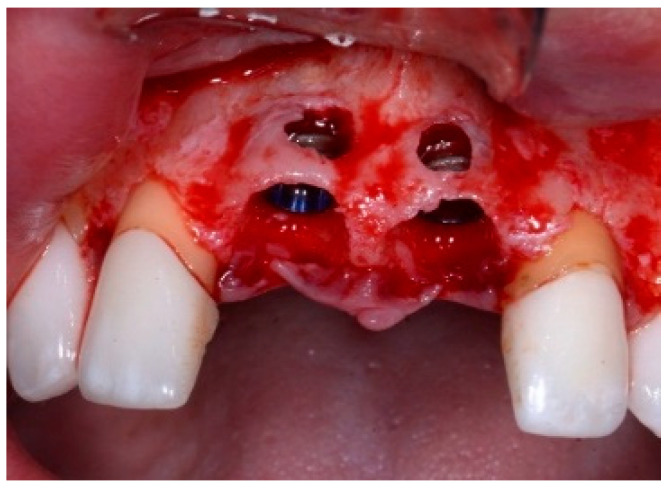
Clinical view of the placement of two maxillary anterior implants.

One hour before the surgical procedure, the patient received a prophylactic dose of 1 g of amoxicillin. Following local anesthesia, surgical access was obtained by raising a full-thickness aesthetic flap extending to the distal line angles of the lateral incisors. The full-thickness flap was extended 3–5 mm beyond the apical margin of the defect. The two central incisor roots were extracted, and the sockets were curetted with surgical curettes to remove all the granulation tissue (Figure 2). The sockets were assessed for the presence of a dehiscence and or fenestration. Two implants (Adin Dental Implants, Englewood, NJ, 07632, USA) were placed according to the manufacturer’s instructions (Figure 3).

**Figure 4:**
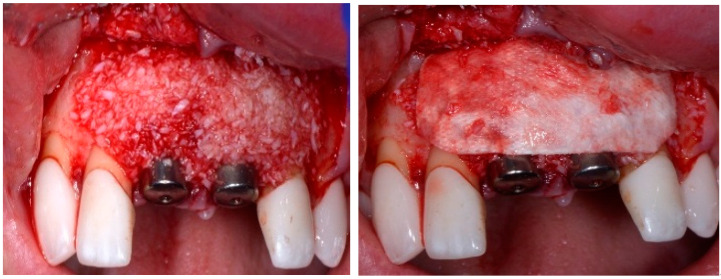
Clinical view post graphing.

**Figure 5:**
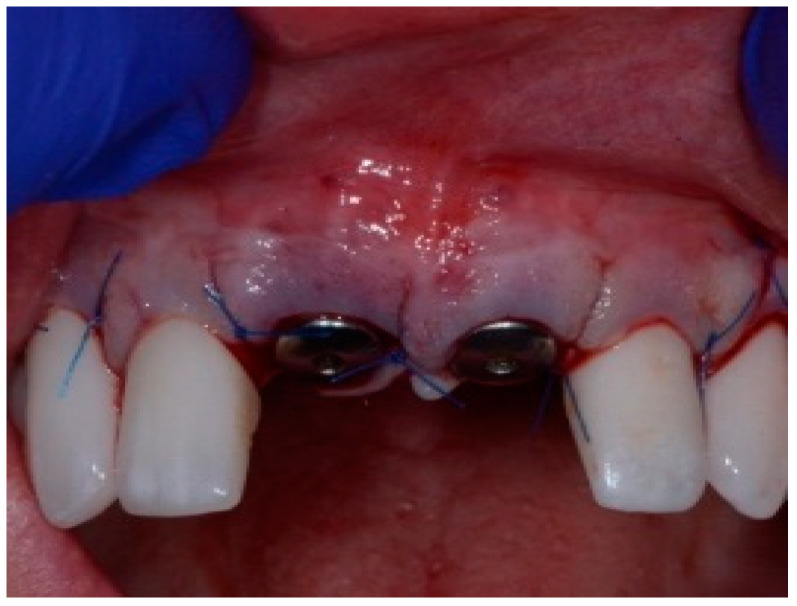
Clinical view of the final flap closure.

Before the graft and membrane is placed over the defect, a periosteal dissection must be performed to release flap tension to facilitate its coronal advancement and closure. The horizontal incision is made 1 mm deep into the periosteum, apical to the mucogingival junction, in a single plane along the length of the flap, connecting the two vertical releasing incisions. After the placement of this shallow incision, a micro-elevator is used to stretch within the incision line. This stretching not only releases the tension within the flap but also allows a more coronal advancement of the flap margin for eventual primary closure, minimizing postoperative incision line opening. This important step in flap management is performed before the placement of the graft material because the periosteal-releasing incision is associated with increased bleeding, which will complicate the graft, the membrane placement, and stabilization. A composite graft material consisting of allograft and xenograft in a 50/50 mix was placed in the gap between the implant and the socket wall over-contouring the facial wall defect (Figure 4). Two healing abutments were placed on the implants, followed by a bioabsorbable collagen membrane (Zmatrix, Osteogenics, Lubbock, TX 79424, USA) placed over the surgical area, which was closed with 5.0 polypropylene sutures (Figure 5). An Essex appliance, fabricated inhouse using a standard suck-down process, was inserted postsurgically. The implants were allowed to heal in a non-submerged environment for a period of 6 months.

### 2.2. Postoperative Management

After the surgical procedure, antibiotic therapy (amoxicillin 500 mg, three times/day) was started and maintained for seven days. Anti-inflammatory and analgesics were prescribed for seven days. The use of 0.12 percent chlorhexidine oral rinses twice a day was indicated during the first two weeks. The patient was seen on a weekly basis for four weeks. 

### 2.3. Follow-Up

**Figure 6:**
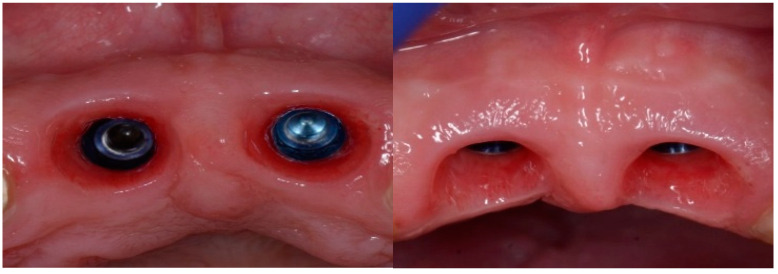
Clinical view of the implant site before final restoration.

**Figure 7:**
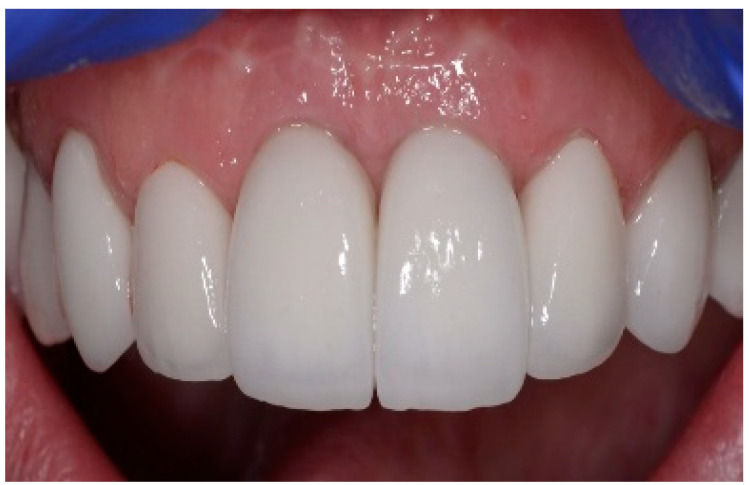
Clinical view of final ceramic crowns on teeth 8 and 9.

Following a 6-month healing period (Figure 6), definitive prosthetic rehabilitation was carried out using zirconia CAD CAM abutments (Straumann, Andover, MA 01810, USA). The ceramic zirconia crowns of teeth 8 and 9 were placed (Figure 7). At the two-year follow-up examination for patients #1 and #3 and three-year for patients #2 and #4, the implants were fully osseointegrated, presenting satisfactory functional and esthetic conditions without clinical or radiographic signs of any pathology.

## 3. Patient 2

**Figure 8:**
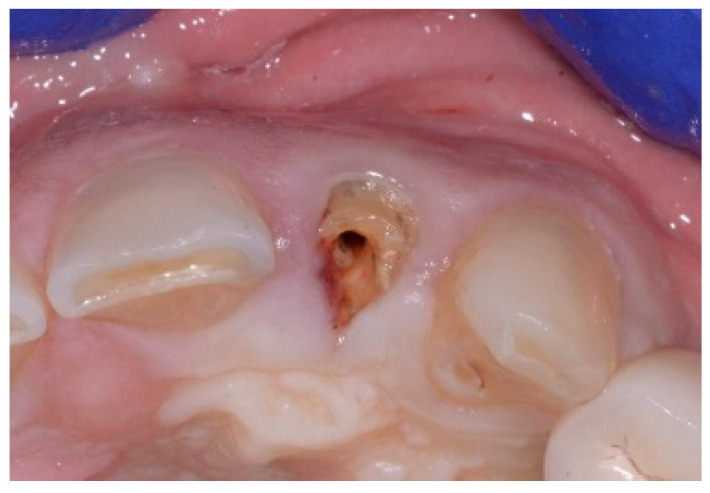
Clinical view of implant placement.

A 51-year-old male in good health and a non-smoker presented to our clinic with a fractured lateral incisor. Clinical and radiographic examination deemed to tooth non-restorable (Figure 8). The treatment plan proposed was to extract the tooth and place an immediate implant with simultaneous bone regeneration of the buccal wall. 

**Figure 9:**
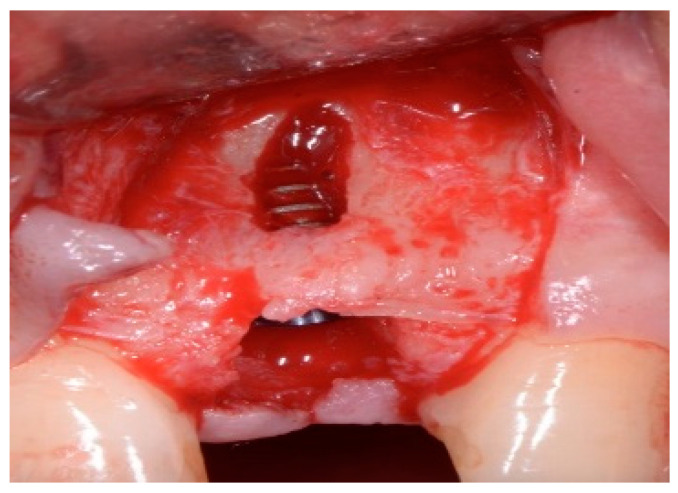
Clinical view of implant replacing.

The patient accepted the proposed treatment plan. The preoperative surgical protocol described in patient 1 was followed. An L-shaped aesthetic flap design was chosen to treat this case. The flap started with an incision extending from the distal line angle of the central incisor to the mesial line angle of the canine. A vertical incision was carried out past the mucogingival junction, followed by the elevation of a full-thickness flap. The remaining tooth root was extracted, maintaining the existing facial bone (Figure 9).

**Figure 10:**
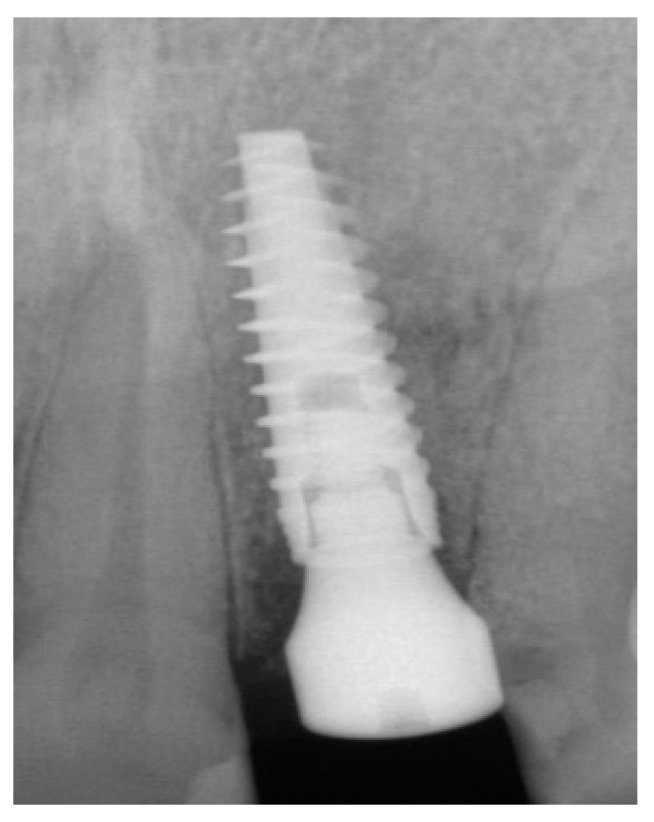
Radiograph of the lateral incisor.

**Figure 11:**
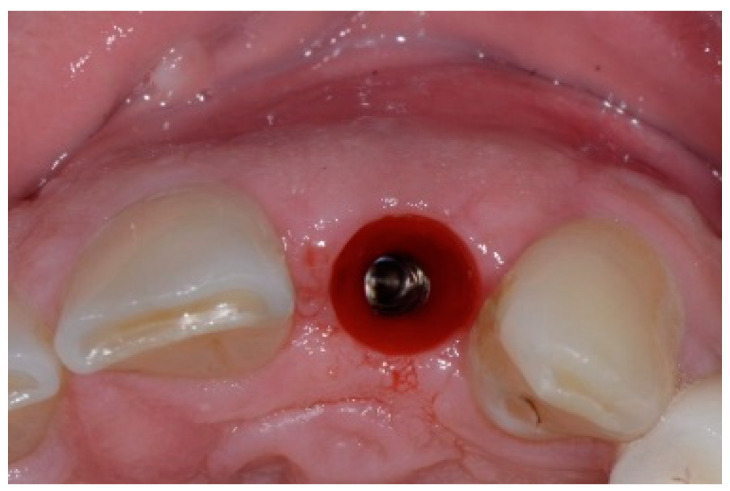
Occlusal view of implant site before final restoration.

**Figure 12:**
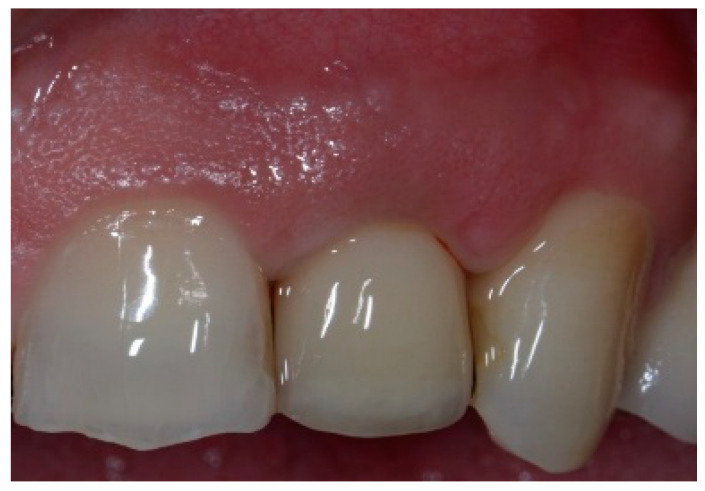
Final implant-supported ceramic crown.

The surgical protocol was followed as described for the previous patient. Likewise, an implant was placed, followed by the placement of bone graft material over the defect and covered with a resorbable membrane. Six months later, the implant was fully intergraded (Figure 10), and the soft tissue appeared to be within normal limits (Figure 11). The implant was restored with a ceramic zirconia crown. Figure 12 presents the patient three years after treatment.

## 4. Patient 3

**Figure 13:**
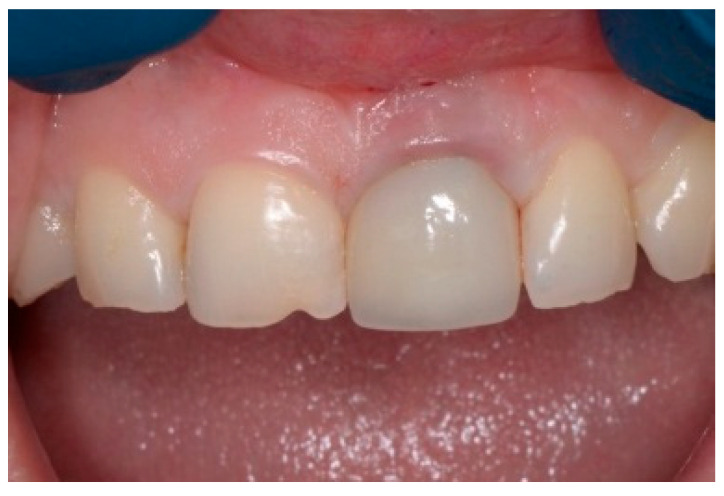
Clinical view of extraction site (note tissue above tooth #9).

**Figure 14:**
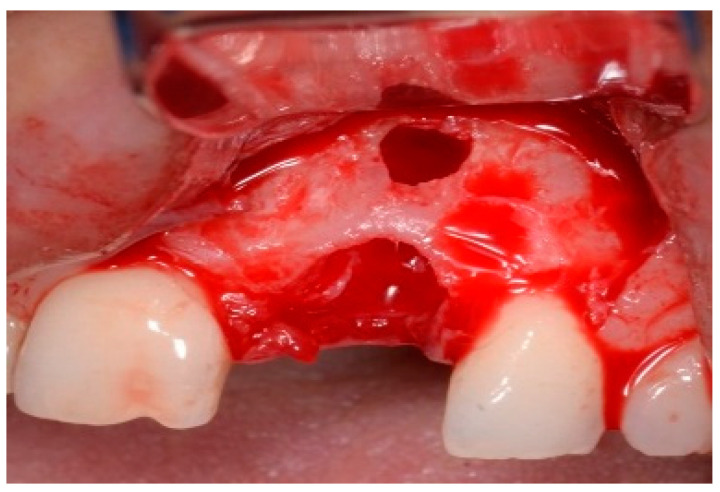
Clinical view of extraction site implant.

**Figure 15:**
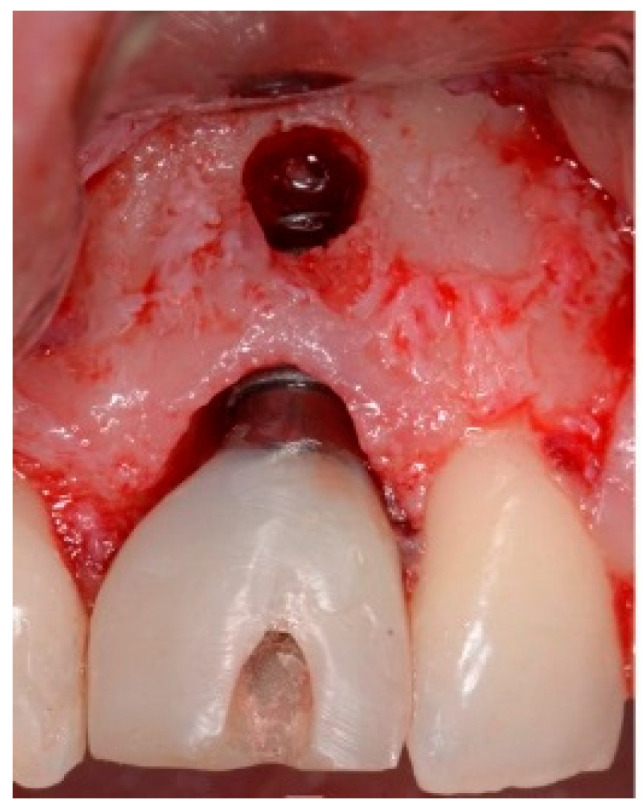
Clinical temporary crown.

**Figure 16:**
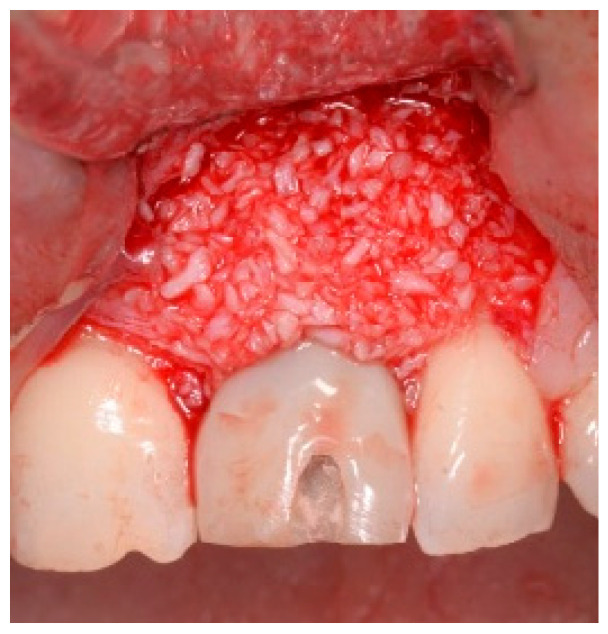
Clinical view post grafting.

**Figure 17:**
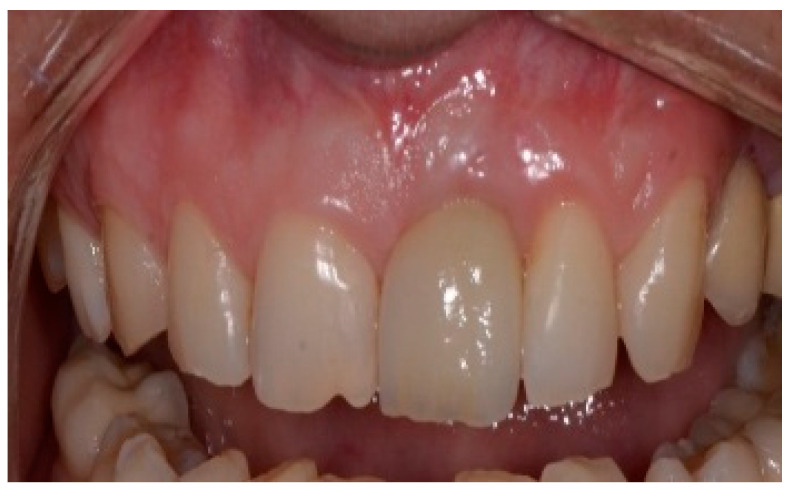
Clinical view of final implant-supported ceramic crown.

A 50-year-old female in good health and a non-smoker presented to our clinic with a failing maxillary left central incisor (Figure 13). The treatment plan consisted of the extraction of the existing tooth and replacing it with an implant. The preoperative surgical protocol described in patient 1 was followed. An L-shaped aesthetic flap design was used, revealing a buccal wall defect (Figure 14). The implant was placed with a screw-retained chair-side temporary crown (Figure 15), followed by the placement of bone graft material over the boney defect (Figure 16). A double-layer collagen membrane was placed and the flap closed. After six months, the final ceramic crown was inserted (Figure 17).

## 5. Patient 4

**Figure 18:**
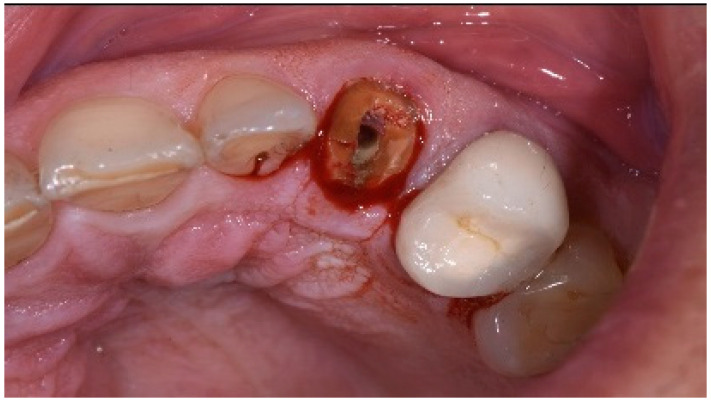
Clinical view of fractured tooth #11 and existing crown on tooth #12.

**Figure 19:**
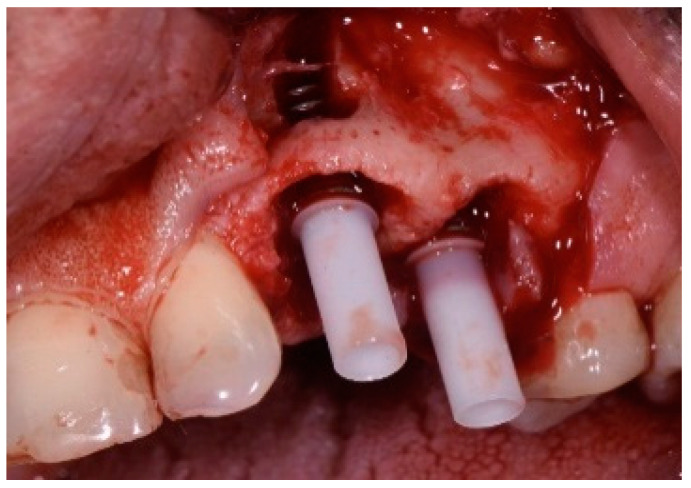
Clinical view of implants and boney defects.

**Figure 20:**
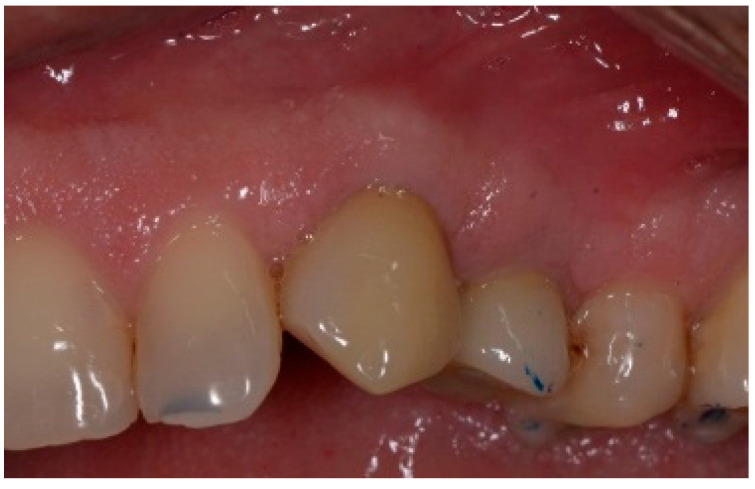
Clinical view of final implant-supported ceramic crowns.

A 51-year-old male patient in good health and a non-smoker presented with a fractured maxillary canine with recurrent subgingival caries, and a failing root canal on the maxillary first premolar (Figure 18). The treatment plan consisted of the extraction of both teeth #11 and #12 roots, followed by the placement of implants. The preoperative surgical protocol described in patient 1 was followed. Figure 19 represents the residual boney defect associated with this area after implant placement. Similar to the previous cases, graft material was placed, and the area was closed for healing. Chairside temporary crowns were fabricated, followed by the placement of zirconia crowns in six months. Figure 20 represents the area three years after treatment.

## 6. Discussion

Over the last several years, IIP has become popular in fresh extraction sockets. IIP not only reduces the treatment time, optimizing the aesthetics, but also improves patient satisfaction. As a result, the IIP has become the treatment of choice in many clinical settings because it simplifies the surgical procedure and provides an instant aesthetic result; it can also maximize the preservation of the bone and soft tissue in the aesthetic zone. Despite the high success and survival rate of IIP [13], the 2018 ITI consensus report suggested that IIP is not a scientifically validated protocol and cases should be selected carefully to reduce the potential risks [14]. In the presence of a damaged alveolus, particularly in the presence of a large buccal dehiscence or fenestration, IIP may be associated with a higher risk of achieving unfavorable aesthetic outcomes. Early implant protocol or ridge preservation at the time of tooth extraction and delayed or late implant placement should be considered [14]. In the event of a larger vertical wall defect present on the facial bone, early implant placement with GBR in a flapped surgical protocol and delayed provisionalization was regarded as a safer approach with a high success rate [15]. This case series proposed a new method for tooth extraction, where flap surgery, immediate implant placement, and GBR were completed in a single surgical session at sites with a large buccal wall deficiency, aiming to simplify the treatment protocol. In the present study, immediate implant placement in sites with chronic periapical pathology and a fenestration defect of the buccal wall resulted in an excellent aesthetic outcome. Some clinical reports have suggested that a history of endodontic or periodontal infections is a predictive risk marker for future implant infection and failure [16,17]. This hypothesis may be justified by the possibility of soft and hard tissue contamination located near the implant surgical bed. This has led most clinicians to avoid immediate placement of dental implants at infected sites and to consider periapical infection a contraindication for immediate implant placement. On the other hand, the placement of immediate implants in chronically infected sites may have successful outcomes and is not a contraindication in all cases. A very stringent protocol option to achieve a successful outcome was proposed by Novaes Jr and Novaes in 1998 [18], consisting of the elimination of the etiological factors and the creation of favorable conditions for tissue healing. In the first step, the patient must receive oral hygiene instructions including scaling and root planning to perform good plaque control. After one week, a reduction in soft tissue inflammation can be noted, and the surgery in association with the use of antibiotics can be performed. The contaminated soft and hard tissue removal by meticulous debridement combined with pre- and postoperative antibiotics will establish a favorable basis for bone healing and osseointegration. However, some other factors have to be considered: absence of active purulent infection; sufficient bone in the apical and palatal direction to allow the implant to be placed with sufficient primary stability in the correct 3D position; adequate gingival tissue thickness; absence of gingival recession. Preservation of the buccal wall crest will permit stability of the gingival position, avoiding black spaces and gingival recession or implant abutment exposure, giving an optimal aesthetic result. The four patients obtained good primary wound healing without any infection or wound exposure. The four cases presented followed a stringed surgical protocol, achieving a success with acceptable aesthetic outcomes. 

Aesthetic implant restorations are defined as those that resemble natural teeth in different aspects [16]. The placement of an implant prosthesis in the maxillary anterior region is particularly challenging due to the high esthetic demands and complex anatomical considerations. Several factors influence the esthetic outcome of immediate implant placement. These risk factors should be thoroughly discussed with the patient before initiating treatment to manage expectations and prevent post-treatment dissatisfaction. Esthetic risk factors can be classified into two main categories: clinician-related factors and patient-related factors. Clinician-related factors included improper implant placement (position and angulation), improper selection of implant diameter, and poor soft and hard grafting techniques. Patient-related factors include the periodontal phenotype (thin tissue, deficient facial bone, and soft tissue), presence of infection, interproximal bone height, previous adjacent restorations, edentulous space size (mesio-distal dimension of the extraction site), and previous surgeries. Despite following the established surgical protocol for immediate implant placement—such as positioning implants in a lingual position along the long axis of the adjacent tooth, maintaining a 2 mm gap from the socket wall, placing implants 1.5 mm below the bone crest or 3 mm below the free gingival margin, and using a slow-resorbing bone graft—the esthetic outcomes in two of the presented clinical cases (Case 1 and Case 3) were clinically acceptable but not ideal. The aesthetic outcomes of these two cases were negatively affected by (a) the presence of scar tissue due to repeated periodontitis (crown lengthening and apicoectomy), (b) the uneven gingival margin, (c) a wide interdental space, (d) the interproximal space height > 5 mm (crest of the bone to contact point), and (e) prosthetic design.

These findings emphasize the need for careful preoperative assessment and meticulous surgical and prosthetic planning to achieve optimal esthetic outcomes in anterior maxillary implant restorations.

In each of these four patient examples, the patients were presented with individual treatment plans and advised that oral photographs of their treatment may be used for publication.

The patients were assured that no identifying information would be used and that no photographs would reveal any identifiable features, tattoos, or other markings. Each patient gave their permission. Each patient was very pleased with the outcome and the esthetics of the soft tissue and restorations.

## 7. Conclusions

The clinical soft tissue outcomes revealed that the midfacial gingival margin had minimal or no recession at two years. This indicated that the lingual implant placement combined with the GBR procedure led to the formation of a new regenerated buccal wall that functioned as a stable new buccal plate, preventing midfacial recession from occurring. Key aspects such as proper patient selection, surgical incision, flap design, tension-free flap advancement, and elaborate suturing were important in avoiding any postoperative complications. Clinicians should utilize this protocol with caution, as the principles discussed in this article cannot be applied to every case. Proper diagnosis, planning, and execution are key factors in achieving predictable results with this protocol. 

## Data Availability

No new data were created or analyzed in this study. It presents the clinical outcomes of a surgical technique.
